# Influence of sheath size on suction mini‐PCNL outcomes: An observational study from the EAU endourology section and the global suction in mini‐PCNL collaborative study group

**DOI:** 10.1002/bco2.70134

**Published:** 2025-12-29

**Authors:** Vineet Gauhar, Bhaskar Somani, Steffi Kar‐Kei Yuen, Kemal Sarica, Marek Zawadzki, Abhishek Singh, Khi Yung Fong, Angelo Cormio, Wei Zhu, Jaisukh Kalathia, Nariman Gadzhiev, Vigen Malkhasyan, Yadgar Shwani, Oriol Angerri Feu, Ben H. Chew, Guohua Zheng, Thomas R. W. Herrmann, Daniele Castellani

**Affiliations:** ^1^ Endourology Section of the European Association of Urology Arnhem The Netherlands; ^2^ Department of Urology Ng Teng Fong General Hospital Singapore Singapore; ^3^ Department of Urology Asian Institute of Nephrology and Urology Hyderabad Telangana India; ^4^ Department of Urology University Hospital Southampton NHS Trust Southampton UK; ^5^ Department of Surgery, S.H. Ho Urology Centre The Chinese University of Hong Kong Hong Kong China; ^6^ Department of Urology, School of Medicine Biruni University İstanbul Turkey; ^7^ Urology Unit St. Anna Hospital Piaseczno Poland; ^8^ Urology Unit Muljibhai Patel Urological Hospital Nadiad Gujarat India; ^9^ Department of Urology Sengkang General Hospital Singapore Singapore; ^10^ Department of Urology and Renal Transplantation, Policlinico Riuniti di Foggia University of Foggia Foggia Italy; ^11^ Urology Unit Azienda Ospedaliero‐Universitaria delle Marche Ancona Italy; ^12^ Department of Urology The First Affiliated Hospital of Guangzhou Medical University Guangzhou China; ^13^ Fortune Urology Clinic Botad Gujarat India; ^14^ Department of Urology Saint‐Petersburg State University Hospital Saint‐Petersburg Russia; ^15^ Department of Urology Moscow Urology Center, Botkin Hospital Moscow Russia; ^16^ Urology Unit Hawler Medical University Erbil Iraq; ^17^ Department of Urology, Fundació Puigvert Autonomous University of Barcelona Barcelona Spain; ^18^ Department of Urology University of British Columbia Vancouver Canada; ^19^ Department of Urology Spital Thurgau AG, Kantonsspital Frauenfeld Frauenfeld Switzerland; ^20^ Department of Surgical Sciences, Division of Urology Stellenbosch University Stellenbosch Western Cape South Africa; ^21^ Hannover Medical School Hannover Germany

**Keywords:** kidney calculi, lithotripsy, percutaneous nephrolithotomy, sheath, suction

## Abstract

**Objective:**

We aim to evaluate the association between sheath size and outcomes in suction mini‐percutaneous nephrolithotomy (SM‐PCNL).

**Materials and Methods:**

A prospective, multicentre study enrolled 1534 patients undergoing SM‐PCNL from March to November 2024 across 30 centres. Patients were stratified into three groups: Group 1 (14–15 Fr, *n* = 780), Group 2 (16–18 Fr, *n* = 388), and Group 3 (20–22 Fr, *n* = 366). Primary outcome was 30‐day stone‐free rate (SFR) determined by non‐contrast CT. Secondary outcomes included complications, operative times and hospital stay.

**Results:**

Group 3 achieved the highest zero residual fragment rate (92.6%) compared to Groups 2 (80.7%) and 1 (79.5%) (*p* < 0.001). Operative times were shortest in Group 3 (36 min) and longest in Group 2 (65 min). Larger sheaths were associated with significantly higher overall complication rates: Group 1 (7.6%), Group 2 (14.4%) and Group 3 (14.8%) (*p* < 0.001). Transfusion requirements increased with sheath size: 0% (Group 1), 1.5% (Group 2) and 3.3% (Group 3). Group 3 had exclusive pleural injuries requiring chest tubes (2.7%) and highest pelvicalyceal perforation rates (4.1% vs 1.3% in Group 2, 0% in Group 1). Larger sheath (16/18 Fr: OR 1.82; 21/22 Fr: OR 4.14) and single step dilation (OR 3.84) were associated with higher odds of zero residual fragments. Sheath size 21/22 Fr (OR 2.12) and increasing Guys stone score (score 2: OR 1.94; score 3: OR 3.51; score 4: OR 2.63 95% CI) were factors significantly associated with higher odds of overall complications.

**Conclusions:**

Sheath selection in SM‐PCNL requires balancing efficacy against safety. Larger sheaths (20–22 Fr) optimize stone clearance but increase complications. Smaller sheaths (14–15 Fr) offer superior safety for simple cases. Intermediate sizes (16–18 Fr) may represent an optimal compromise for moderately complex stones.

## 1 INTRODUCTION

Mini‐percutaneous nephrolithotomy (M‐PCNL) has been demonstrated to provide superior perioperative outcomes compared to standard PCNL, with lower invasiveness, improved patient quality of life, and comparable stone‐free rates (SFRs).^1^ Suction M‐PCNL (SM‐PCNL) has gained recognition as an effective approach for the management of kidney stones. The access sheath size in this technique typically ranges from 14 to 22 Fr, a parameter that may significantly influence both operative and perioperative outcomes.^2^

In conventional M‐PCNL, studies have indicated that the outcomes of different access sizes were comparable for stones up to 25 mm, but the surgical time was significantly shortened with the increase of size.^3^ However, such comparative data remain limited for SM‐PCNL.

SM‐PCNL may further improve the M‐PCNL experience by reducing intrarenal pressure (IRP), enhancing fragment evacuation and improving visibility, factors which have contributed to its increasing adoption.^4^ The efficacy of fragment evacuation in SM‐PCNL relies on both active suction and the Venturi effect. The latter utilizes Bernoulli's principle: as irrigation fluid passes through the narrow access sheath, velocity increases and pressure drops, creating a low‐pressure environment that facilitates removal of stone fragments.^5^ Theoretically, narrower sheaths enable faster irrigation and potentially quicker evacuation; however, due to the limited volume of the pelvicalyceal system when occupied by a large stone, practical observations indicate that operative time is inversely related to sheath diameter. Larger sheaths enhance the effectiveness of active suction, allowing more rapid and efficient clearance of larger stone fragments and debris, while minimizing the risk of dispersing fragments into other calyces.^6^ Hence, it is not well understood if there is a best sheath size that surgeons can use for all SM‐PCNL or should choice of the sheath size be tailored and individualized to suit specific cases. Surgeons often have personal preferences regarding sheath type and size.

We hypothesized that larger sheath sizes in SM‐PCNL would achieve higher SFRs due to enhanced suction efficiency and the ability to remove larger fragments. Hence, our study aimed to determine the association between sheath size and 30‐day perioperative outcomes aiming to offer insights into the advantages and disadvantages of using different sheath sizes in SM‐PCNL.

## 2 METHODS

This was a prospective, multicentre, observational study. Patients were enrolled between March 2024 to November 2024 by 21 surgeons from 30 centres. Patient consent was obtained to contribute to the IRB‐approved STUMPS registry by the Asian Institute of Nephro‐Urology after ethical board clearance (protocol: AINU #01/2024) maintained by the principal site. The full study protocol has been previously published.^7^ Briefly, inclusion criteria were patients aged ≥18 years who underwent prone or supine SM‐PCNL for renal stones. Patients who underwent non‐suction PCNL or endoscopic combined intra renal surgery or had insufficient data obtainable from patient records were excluded.

The SM‐PCNL procedure was done using single‐use or reusable percutaneous suction access nephrostomy sheath (14–22 Fr) with any energy device as per centre availability. Sheaths used were disposable sheaths of 14 Fr, 16 Fr and 18 Fr Clearpetra (Wellead, Panyu, Guangzhou); 15 Fr, 18 Fr, 21 Fr Shah sheath; 16 Fr, 18 Fr and 20 Fr from Seplou medical (Hangzhou, China) and 16 Fr from Reborn medical (London, UK). Reusable sheaths were Hawk medical 16 Fr (Shenzhen, Guangdong, China), Urologeman sheath 18 Fr and 21 Fr Shah metal sheath.

For this analysis, patients were divided into three groups according to sheath size: Group 1: 14/15 Fr; Group 2: 16/18 Fr; Group 3: 21/22 Fr. The choice of energy source, sheath size, patient positioning, puncture and tract dilation methods, and postoperative exit strategy were at the respective surgeons' discretion based on their experience and available resources. All positive preoperative cultures were treated as per bacterial sensitivities, but a negative urine culture preoperatively was not mandatory. Anticoagulants or antiplatelets were stopped 3 days prior and restarted as per the surgeon's discretion. As this was a real‐world study, surgeons were allowed to perform SM‐PCNL as per their practice but respecting inclusion and exclusion criteria. Surgeons were asked to grade their experience of suction use at the end of each case using a 5‐point Likert‐type scale (1 = *excellent*; 2 = *very good*; 3 = *good*; 4 = *average*; 5 = *difficult*). Pain score was assessed after 12 h and within 24 h after surgery using a 10‐point visual analogue scale where 1 was the lowest and 10 being the highest score administered. Complications within 30 days of surgery were graded according to Clavien scoring system modified for PCNL.^8^

All patients were monitored for 30 days, with any readmissions for re‐intervention or complications recorded. The main outcome was SFR, determined by a non‐contrast CT using bone window 30 days post‐PCNL. Stone‐free status was classified as follows: *Grade A*: 100% stone free, indicating zero residual fragments (RF); *Grade B*: single RF measuring up to 4 mm in maximum diameter; *Grade C*: single RF > 4 mm in maximum diameter or multiple RFs of any size.

### 2.1 Statistical analysis

Continuous variables were described using median and interquartile range, while categorical variables were described using absolute numbers and percentages. To compare variables between groups, the χ^2^ test was applied for categorical variables and the Kruskal‐Wallis test for continuous variables. Finally, two a priori multivariable logistic regression analyses were conducted to identify independent predictors of Grade stone‐free status and overall complications. Variables included in the models were selected according to variables suggested in previous literature to impact SFR and complications. Results are expressed as odds ratios (OR) with 95% confidence intervals (CI) and corresponding p‐values.

All statistical analyses were performed using R Statistical language, version 4.3.0 (R Foundation for Statistical Computing, Vienna, Austria) with *p* < 0.05 indicating statistical significance.

## 3 RESULTS

A total of 1534 patients were included and divided into Group 1 (14–15 Fr, *n* = 780), Group 2 (16–18 Fr, *n* = 388), and Group 3 (20–22 Fr, *n* = 366) (Table 1). The median age was comparable ranging from 49 to 52 years (*p* = 0.09). Significant differences in gender distribution were observed, with Group 1 having the lowest proportion of male patients (36.2%) compared to Groups 2 and 3 (46.4% and 46.2%, respectively; *p* < 0.001).

**TABLE 1:** Patients' baseline characteristics.

	Group 1 (*N* = 780)	Group 2 (*N* = 388)	Group 3 (*N* = 366)	p
Age, years, median [IQR]	49 [38, 60]	51 [39, 62]	52 [41, 60]	0.09
Male gender, *n* (%)	282 (36.2)	180 (46.4)	169 (46.2)	**<0.001**
ASA score, *n* (%)				
1	411 (52.7)	183 (47.2)	194 (53.0)	**0.001**
2	312 (40.0)	153 (39.4)	131 (35.8)	
3	51 (6.5)	52 (13.4)	41 (11.2)	
4	6 (0.8)	0	0	
Body mass index, kg/m^2^, median [IQR]	27.0 [24.0, 29.5]	26.2 [23.4, 31.0]	25.5 [23.0, 28.0]	**<0.001**
Diabetes, *n* (%)	125 (16.0)	87 (22.4)	90 (24.6)	**0.001**
Presentation, *n* (%)				
Hematuria	66 (8.5)	40 (10.3)	5 (1.4)	**<0.001**
Pain	572 (73.3)	245 (63.1)	308 (84.2)	
Fever	28 (3.6)	25 (6.4)	22 (6.0)	
Incidental	114 (14.6)	78 (20.1)	31 (8.5)	
First time stone former, *n* (%)	582 (74.6)	263 (67.8)	294 (80.3)	**<0.001**
Laterality, *n* (%)				
Left	369 (47.3)	191 (49.2)	184 (50.3)	**<0.01**
Right	364 (46.7)	170 (43.8)	178 (48.6)	
Bilateral	47 (6.0)	27 (7.0)	4 (1.1)	
Guy's stone score, *n* (%)				
1	447 (57.3)	146 (37.6)	209 (58.2)	**<0.001**
2	235 (30.1)	114 (29.4)	100 (27.9)	
3	72 (9.2)	79 (20.4)	43 (12.0)	
4	26 (3.3)	49 (12.6)	7 (1.9)	
Hounsfield units, median [IQR]	1167 [920, 1369]	1100 [800, 1336]	1235 [1050, 1362]	**<0.001**
Largest stone diameter, cm, median [IQR]	1.9 [1.5, 2.7]	2.2 [1.7, 3.0]	1.9 [1.6, 2.3]	**<0.001**
Stone volume, mm^3^, median [IQR]	1403 [734, 2665]	1948 [985, 3745]	1850 [1263, 3800]	**<0.001**
Stone location, *n* (%)				
Upper pole	117 (16.9)	32 (12.9)	58 (16.4)	**<0.001**
Interporal	298 (43.1)	77 (31.0)	197 (55.6)	
Lower pole	276 (39.9)	139 (56.0)	99 (28.0)	
Previous PCNL, *n* (%)	30 (3.8)	11 (2.8)	8 (2.2)	0.3

*Note*: Group 1 (<16 Fr): 14 Fr and 15 Fr. Group 2: 16 Fr and 18 Fr. Group 3 (20–22 Fr): 20 Fr, 21 Fr, and 22 Fr.

Abbreviations: ASA, American Society of Anesthesiologists; PCNL, percutaneous nephrolithotripsy.

Comorbidity profiles differed significantly. Group 3 patients had the lowASA scores >2 (47.0%) compared to Groups 1 and 2 (47.3% and 52.8%, respectively; *p* = 0.001). Diabetes mellitus rates were highest in Group 3 (24.6%) compared to Groups 1 and 2 (16.0% and 22.4%, respectively; *p* = 0.001).

When evaluating stone burden and complexity using Guy's stone score (GSS), Group 1 exhibited the highest proportion of GSS1 stones (57.3%), whereas Group 3 had a greater proportion of complex stones (GSS 3: 12.0% versus 9.2% in Group 1; *p* < 0.001). The largest median stone diameter was observed in Group 2 (2.2 cm [1.7, 3.0]), followed by Group 1 (1.9 cm [1.5, 2.7]) and Group 3 (1.9 cm [1.6, 2.3]; *p* < 0.001). Stone volume reached its peak in Group 2 (1948 mm^3^ [985, 3745]) in comparison to Groups 1 and 3 (1403 mm^3^ [734, 2665] and 1850 mm^3^ [1263, 3800], respectively; *p* < 0.001). There were significant differences in the distribution of stone location (*p* < 0.001), with lower pole stones most frequently found in Group 2 (56.0%). Hounsfield units were highest in Group 3 (1235 [1050, 1362]), relative to Groups 1 and 2 (1167 [920, 1369] and 1100 [800, 1336], respectively; *p* < 0.001).

As detailed in Table 2, the supine position was employed most frequently in Group 1 (71.9%) compared to Groups 2 and 3 (50.0% and 34.4%, respectively; *p* < 0.001). Spinal anaesthesia was most prevalent in Group 3 (61.5%) versus Groups 1 and 2 (42.9% and 35.6%, respectively; *p* < 0.001). Significant variation was observed in puncture modality (*p* < 0.001); fluoroscopy‐only guidance was predominant in Group 3 (97.3%), while combined fluoroscopy and ultrasound guidance was more common in Groups 1 and 2 (27.1% and 38.9%, respectively). Supracostal access occurred most frequently in Group 3 (38.8%) as opposed to Groups 1 and 2 (27.9% and 18.8%, respectively; *p* < 0.001). Notable differences in tract dilation methods were evident across groups (*p* < 0.001), with single‐step dilation favoured in Groups 1 and 2 (60.8% and 69.8%), and serial dilation with metal dilators predominating in Group 3 (57.1%). Utilization of mechanical lithotripsy correlated with sheath size, being most frequent in Group 3 (79.8%), followed by Group 2 (25.3%) and Group 1 (10.4%; *p* < 0.001). Stone basket extraction was required most often in Group 3 (36.6%) when compared to Groups 1 and 2 (21.2% and 7.7%, respectively; *p* < 0.001).

**TABLE 2:** Procedural characteristics.

	Group1 (*N* = 780)	Group 2 (*N* = 388)	Group3 (*N* = 366)	p
Spinal anaesthesia, *n* (%)	335 (42.9)	138 (35.6)	225 (61.5)	**<0.001**
Supine position, *n* (%)	561 (71.9)	194 (50.0)	126 (34.4)	**<0.001**
Puncture modality, *n* (%)				
Fluoroscopy only	515 (66.0)	227 (58.5)	356 (97.3)	**<0.001**
Ultrasound only	40 (5.1)	3 (0.8)	0	
Fluoroscopy + ultrasound	211 (27.1)	151 (38.9)	6 (1.6)	
Endoscopy guided	14 (1.8)	7 (1.8)	4 (1.1)	
Disposable sheath, *n* (%)	528 (67.7)	227 (60.1)	217 (59.3)	**<0.01**
Supracostal access (above 11th rib), *n* (%)	218 (27.9)	73 (18.8)	142 (38.8)	**<0.001**
Tract dilation method, *n* (%)				
Serial with metal dilators	220 (28.2)	33 (8.5)	209 (57.1)	**<0.001**
Serial with non‐metal dilators	77 (9.9)	84 (21.6)	14 (3.8)	
Balloon	9 (1.2)	0	31 (8.5)	
Single step dilatation	474 (60.8)	271 (69.8)	112 (30.6)	
Mechanical lithotripsy, *n* (%)	81 (10.4)	98 (25.3)	292 (79.8)	**<0.001**
Basket required for stone extraction, *n* (%)	165 (21.2)	30 (7.7)	134 (36.6)	**<0.001**
Lithotripsy time, min, median [IQR]	18 [11, 27]	31 [16, 50]	9.0 [5.0, 17]	**<0.001**
Total operation time, min, median [IQR]	43 [30, 71]	65 [40, 95]	36 [26, 51]	**<0.001**
Exit strategy, *n* (%)				
Nephrostomy tube only	186 (23.8)	41 (10.6)	20 (5.5)	**<0.001**
Tubeless with ureteral stent	458 (58.7)	176 (45.4)	121 (33.1)	
Tubeless with overnight ureteric catheter	18 (2.3)	94 (24.2)	15 (4.1)	
Nephrostomy tube + ureteral stent	108 (13.8)	68 (17.5)	210 (57.4)	
Totally tubeless	10 (1.3)	9 (2.3)	0	

*Note*: Group 1 (<16 Fr): 14 Fr and 15 Fr. Group 2: 16 Fr and 18 Fr. Group 3 (20–22 Fr): 20 Fr, 21 Fr, and 22 Fr.

Significant differences in operative time were observed across groups (*p* < 0.001). Group 3 achieved the shortest median operative time of 36 min [IQR 26–51], followed by Group 1 at 43 min [IQR 30–71], while Group 2 required the longest time at 65 min [IQR 40–95]. Notably, Group 2 patients had the largest median stone diameter and stone volume, which likely contributed to the extended operative duration. The shorter operative time in Group 3, despite managing complex stones, can be attributed to enhanced fragment evacuation through larger sheath diameter.

Complication rates differed significantly, with Group 1 showing the lowest (7.6%) compared to Groups 2 (14.4%) and 3 (14.8%) respectively (Table 3). Intraoperative bleeding was least in Group 1 (89.1% had none), while blood transfusion needs (Clavien grade 2) increased with sheath size: 0% (Group 1), 1.5% (Group 2) and 3.3% (Group 3). Pelvicalyceal perforations requiring nephrostomy (Clavien grade 3a) occurred only in Groups 2 (1.3%) and 3 (4.1%). Pleural injuries needing chest tubes (Clavien grade 3a) were exclusive to Group 3 (2.7%). There was one case of sepsis (Clavien grade 4) in each group. Group 2 reported the highest median postoperative pain scores (3.0 [2.0, 4.0]) and longest hospital stays (3 days vs. 2 days for others, *p* < 0.001 for both respectively).

**TABLE 3:** Intraoperative and postoperative outcomes.

	Group 1 (*N* = 780)	Group 2 (*N* = 388)	Group 3 (*N* = 366)	*p*
Intraoperative bleeding after dilatation, *n* (%)				
No bleeding	695 (89.1)	300 (77.3)	309 (84.4)	**<0.001**
Case abandoned	1 (0.1)	4 (1.0)	0	
Blood transfusion (Clavien 2), *n* (%)	0	6 (1.5)	12 (3.3)	**<0.001**
Bleeding managed by angioembolisation (Clavien 3b), *n* (%)	1 (0.1)	0	0	0.62
Renal pelvic perforation managed by prolonged nephrostomy tube or postoperative placement of nephrostomy (Clavien 3a), *n* (%)	0	5 (1.3)	15 (4.1)	**<0.001**
Colon perforation managed conservatively using intravenous fluid and antibiotics (Clavien 2), *n* (%)	2 (0.3)	0	0	0.38
Pneumothorax managed by intercostal draining under local anaesthesia (Clavien 3a)	0	0	10 (2.7)	**<0.001**
Postoperative pain score	2.0 [1.0, 3.0]	3.0 [2.0, 4.0]	2.0 [1.0, 3.0]	**<0.001**
Infectious complications, *n* (%)				
None	717 (91.9)	310 (79.8)	336 (91.8)	**0.02**
Postoperative fever (>38.0°C) managed by observation without antibiotics (Clavien 1)	50 (6.4)	43 (11.1)	20 (5.5)	
Sepsis needing intensive care unit admission (Clavien 4)	1 (0.1)	1 (0.3)	1 (0.3)	
Postoperative fever (>38.0°C) managed with antibiotics in the ward (Clavien 2), *n* (%)	12 (1.5)	34 (8.8)	9 (2.5)	
Postoperative Stone‐free status on 30‐day CT scan, *n* (%)				
Grade A: zero RF	620 (79.5)	313 (80.7)	339 (92.6)	**<0.001**
Grade B: single RF ≤ 4 mm	128 (16.4)	37 (9.5)	23 (6.3)	
Grade C: single RF > 4 mm/multiple of any size	32 (4.1)	38 (9.8)	4 (1.1)	
Hospital stay, days, median [IQR]	2 [1, 3]	3 [2, 4]	2 [2, 3]	**<0.001**
Readmission for any reason within 72 h, *n* (%)	6 (0.8)	15 (3.9)	5 (1.4)	**<0.001**
Reintervention after 30 days, *n* (%)	14 (1.8)	20 (5.2)	4 (1.1)	**<0.001**

*Note*: Group 1 (<16 Fr): 14 Fr and 15 Fr. Group 2: 16 Fr and 18 Fr. Group 3 (20–22 Fr): 20 Fr, 21 Fr, and 22 Fr.

Group 2 had the highest rate (3.9%) of 72‐hour readmission compared to Groups 1 and 3 (0.8% and 1.4%, respectively). Similarly, reintervention requirements after 30 days were highest in Group 2 (5.2%) compared to Groups 1 and 3 (1.8% and 1.1%, respectively; *p* < 0.001).

Thirty‐day SFRs were significantly different across groups (*p* < 0.001) (Table 3). Group 3 achieved the highest Grade A (92.6%), followed by Group 2 (80.7%) and Group 1 (79.5%). Grade B stone‐free status was highest in Group 1 (16.4%) than Groups 2 and 3 (9.5% and 6.3%, respectively). Grade C was highest in Group 2 (9.8%) compared to Groups 1 and 3 (4.1% and 1.1%, respectively).

Table 4 shows multivariable analysis of factors affecting Grade A stone‐free status. Using larger sheath (16/18 Fr: OR 1.82 95% CI 1.25–2.70, *p* = 0.01; 21/22 Fr: OR 4.14 95% CI 2.48–7.15, *p* < 0.001) and single step dilation (OR 3.84 2.46–6.03, *p* < 0.001) were associated with higher odds of Grade A stone‐free status. Sheath size 21/22 Fr (OR 2.12 95% CI 1.28–3.52, *p* = 0.01) and increasing GSS (score 2: OR 1.94 95% CI 1.26–3.00, *p* = 0.01; score 3: OR 3.51 95% CI 2.14–5.74, *p* < 0.001; score 4: OR 2.63 95% CI 1.22–5.44, *p* = 0.01) were factors significantly associated with higher odds of overall complications (Table 5).

**TABLE 4:** Logistic regression analysis of factors associated with grade a stone‐free status.

	Odds ratio	95% confidence interval	*p*
Sheath size (reference Group 1)			
Group 2	1.82	1.25–2.70	**0.002**
Group 3	4.14	2.48–7.15	**<0.001**
Age	0.99	0.98–1.00	0.24
Guy's stone score (reference 1)			
2	0.99	0.98–1.00	0.24
3	0.64	0.44–0.93	**0.02**
4	0.29	0.19–0.46	**<0.001**
Supine position (reference prone)	0.96	0.94–0.98	**<0.001**
Tract dilation method (reference serial with metal dilators)			
Serial with non‐metal dilators	0.53	0.32–0.86	**0.011**
Balloon	2.92	0.91–11.67	0.09
Single step dilatation	3.84	2.46–6.03	**<0.001**
Disposable sheath (reference reusable sheath)	1.09	0.78–1.51	0.62

*Note*: Group 1 (<16 Fr): 14 Fr and 15 Fr. Group 2: 16 Fr and 18 Fr. Group 3 (20–22 Fr): 20 Fr, 21 Fr, and 22 Fr.

**TABLE 5:** Logistic regression analysis of factors associated with overall complications.

	Odds ratio	95% confidence interval	*p*
Sheath size (reference group 1)			
Group 2	1.32	0.84–2.07	0.23
Group 3	2.12	1.28–3.52	**0.01**
Age	1.01	1.00–1.02	0.24
Guy's stone score (reference 1)			
2	1.94	1.26–3.00	**<0.01**
3	3.51	2.14–5.74	**<0.001**
4	2.63	1.22–5.44	**0.01**
Stone volume	1.01	1.00–1.03	0.11
Supine position (reference prone)	0.72	0.47–1.10	0.13
Tract dilation method (reference serial with metal dilators)			
Serial with non‐metal dilators	1.64	0.84–3.17	0.14
Balloon	0.9	0.30–2.30	0.83
Single step dilatation	1.04	0.61–1.81	0.89
Disposable sheath (reference reusable sheath)	0.74	0.51–1.07	0.11

*Note*: Group 1 (<16 Fr): 14 Fr and 15 Fr. Group 2: 16 Fr and 18 Fr. Group 3 (20–22 Fr): 20 Fr, 21 Fr, and 22 Fr.

## 4 DISCUSSION

### 4.1 Influence of sheath size on patient, stone characteristics and surgical times

In this real‐world study involving surgeons with various levels of experience across diverse healthcare systems, results indicate that patient and stone characteristics differed among the three sheath size groups. This observation suggests that sheath size selection may be influenced by pre‐operative assessments. Group 3 sheaths were more frequently used for patients with higher comorbidity rates, raised ASA scores and larger stones (GSS 3 and 4), while smaller sheaths were typically chosen for cases involving smaller stones, better health status and easier access. This approach could aim at minimizing operative time and anaesthesia exposure, which may impact the likelihood of achieving single‐stage SFR. These findings are consistent with the reported operative times and multivariate analysis indicating optimal Grade A stone‐free status in Group 3.

Published literature supports these observations. For example, Yu et al. conducted a randomized trial comparing non‐suction M‐PCNL with 16 Fr, 18 Fr, 20 Fr or 22 Fr accesses, finding no significant difference in surgical outcomes between sheath sizes, but shorter operation times with larger sheaths.^3^ No marked difference was observed in operative time between 20 Fr and 22 Fr. The present study utilized vacuum aspiration, associated with faster performance compared to non‐suction PCNL, and found a median operative time of 36 min, less than Yu study's mean of 43.3 ± 13.1 min. Although there is limited literature regarding optimal surgical times for different sheath sizes, consensus from the International Alliance of Urolithiasis suggests that mini‐PCNL with suction enables effective management of medium‐sized stones (<4 cm) within safe operational thresholds of 60–90 min, not exceeding 120 min.^2^

Overall, the present data evidences a rise in complications with larger sheaths, indicating that the advantages of high‐flow suction must be carefully weighed against heightened risks of tissue trauma, bleeding and the pursuit of a stone‐free status. Lahme et al. also reported that smaller access sizes resulted in longer operative times for fragment clearance.^9^ In our study, increased use of mechanical lithotripsy was noted in Group 3, potentially contributing to reduced lithotripsy and overall operative times. This was in line with a recent meta‐analysis showing that non‐laser devices in PCNL achieved higher SFR than holmium laser PCNL.^10^ Additionally, data from our study indicate that Group 2 patients had the largest median stone diameter and volume, as well as a greater proportion of lower pole stones. This may suggest that a 16–18 Fr sheath is suitable for moderately large and complex stones, providing a middle ground in SM‐PCNL. Variables such as surgeon preference, instrument availability and feasibility of single‐step dilation may also affect sheath selection. International guidelines recommend sheath sizes of 14–18 Fr for stones under 4 cm, although specific recommendations for SM‐PCNL remain absent.^2^

Recent studies by Yang et al.^11^ and Du et al.^12^ demonstrated the effectiveness of advanced vacuum or SM‐PCNL with 18 Fr sheaths in managing renal pelvic pressure and facilitating fragment extraction, including for staghorn calculi, though multiple tracts may be necessary for inaccessible stones. A recent global survey found that 33.4% of surgeons employ SM‐PCNL in all cases, yet many respondents remain uncertain regarding optimal technology, sheath size or lithotripsy technique for SM‐PCNL.^13^ The present study did not include IRP measurements, but future research may further clarify how different nephrostomy sheath sizes affect IRP in SM‐PCNL, in comparison with flexible ureteroscopy, where sheath type and size impact IRP.^14^, ^15^ Nizzardo et al. reviewed access sheath calibres in PCNL and noted limited research on IRP.^14^ The 18 Fr Clearpetra suction system consistently yielded low median IRP (1.8 ± 0.9 mmHg^12^ and 4.1 ± 1.8 mmHg^16^), which are below the 30 mm threshold commonly associated with infectious complications.^17^ Nevertheless, the available evidence from this study does not allow definitive conclusions about how sheath size affects IRP in SM‐PCNL but it is important to consider this as it has a direct bearing on infectious complications.

### 4.2 Impact on and procedural outcomes and complications

The selection of sheath size substantially influenced various procedural outcomes in our study. The data delineates a clear balance between efficacy and safety associated with different sheath calibres. The SFR was highest in Group 3 (92.6%), followed by Group 2 (80.7%) and Group 1 (79.5%), indicating that larger sheaths facilitate more comprehensive stone clearance, particularly in complex cases. Regression analysis corroborates these findings, showing that utilization of a sheath greater than 18 Fr is most strongly associated with achieving zero residual fragments. However, increased efficacy is accompanied by higher complication rates. Group 1 experienced the lowest complication rate (7.6%), while Groups 2 and 3 exhibited higher rates (14.4% and 14.8%, respectively). Yet, regression analysis further identifies larger sheath size as an independent risk factor for complications. Larger sheaths were specifically associated with increased intraoperative bleeding and transfusion requirements, predominantly observed in Group 3, which can be explained by greater parenchymal disruption. Pleural injuries necessitating chest tubes occurred exclusively in Group 3, likely due to supracostal access required for complex, high‐laying stones, raising the inherent risk of pleural injury. This finding might be attributed to the technical differences in puncture approach between the groups, where supracostal access procedures could be performed via upper lateral calyx puncture in the supine position, which provides a more favourable anatomical trajectory with greater distance from the pleural reflection in contrast with upper medial calyx puncture in the prone position, which increases the risk of pleural injury due to the closer proximity of the pleura to the medial aspect of the upper pole and the altered anatomical relationships in prone positioning. This observation underscores the importance of puncture site selection and patient positioning in minimizing pleural complications during supracostal access.

These results are consistent with existing literature demonstrating that larger calibre access, multiple punctures, complex anatomy, higher GSS and extended operative times are all correlated with increased bleeding and infectious complications in PCNL procedures, regardless of suction use.^18^ Conversely, a meta‐analysis by Adhoni et al. found that vacuum‐assisted mini‐PCNL had a significantly lower overall complication rate, including reduced sepsis and postoperative fever rates compared with conventional access sheath.^19^ In the present study, only three cases of sepsis were recorded, one in each group and postoperative fever was common across all groups. It is essential to recognize that fluid absorption, even with suction, may occur during lithotripsy and if unregulated, could result in systemic bacteraemia.^20^ Literature emphasizes the importance of thoroughly treated urine cultures, intraoperative antibiotic prophylaxis, surgical expertise to minimize operative time, and use of suction to reduce infectious complications.^21^ Notably, the smaller sheath may not allow for a high‐flow suction system. Whilst this is a less efficient process and increases surgical time, it minimizes the amount of irrigant fluid needed for suction and thus translates into fewer infective complications due to bacteraemia when surgical times are respected. Another reason why surgeons choose smaller sheaths in simpler stone burdens and less complex anatomy.

### 4.3 Exit strategy, recovery and sheath sizes

In our study, Group 1 patients had a smaller stone burden and least stone complexity, and the same translated to lowest complication rates and the most totally tubeless cases, suggesting a more rapid and comfortable recovery. Yet, the converse of this is perhaps why Group 3 had the highest number of cases where a nephrostomy tube and a ureteral stent were both placed. This is probably due to increasing sheath invasiveness that can affect other outcomes of surgery. Ultimately, surgeons' own choices and practice will affect the choice of exit strategy, but from our study, 16 Fr and 18 Fr may be a “sweet‐spot” wherein good perioperative outcomes can increase the surgeon confidence in adopting a tubeless or totally tubeless procedure. Interestingly, in literature, due to the minimally invasive nature of mini‐PCNL, it is theoretically anticipated that there will be reduced frequency for nephrostomy tube usage, and 93.7% surgeons agree with this viewpoint, but 46.9% continued to insert tubes irrespective of tract size.^21^, ^22^, ^23^ This is especially due to concerns of bleeding, subsequent ureteric obstructions or concerns of missed perforations of the pelvic‐calyceal system and retained stones.

### 4.4 Strengths and limitations of the present study

Our study demonstrates that selecting sheath size in PCNL is not a universal process; rather, it requires careful consideration of multiple variables. The heterogeneity of energy sources, patient positioning and exit strategies represents a significant limitation, as these variables may have independently influenced outcomes and complicate the interpretation of results attributed to specific technical modifications in our series. Nevertheless, our findings offer an evidence‐based framework to inform this critical surgical decision‐making, though it is important to note that our work represents a comparative analysis of real‐world practices and lacks the rigor of a randomized controlled trial. However, our results provide valuable insights into current SM‐PCNL approaches, illustrating a clear distinction between the efficacy and safety profiles of various sheath sizes.

For the first time, using a large prospective dataset on SM‐PCNL, we have observed that larger sheaths (20–22 Fr) are associated with higher SFRs and reduced operative times, attributable to the ability to employ more powerful lithotripters and high‐flow suction for efficient stone clearance. Nevertheless, these benefits are counterbalanced by increased complication rates, such as greater bleeding and parenchymal injury. Additionally, factors such as surgeon experience, whether the puncture was performed by the primary surgeon or an assistant, and the use of instruments from different manufacturers may directly impact operative outcomes, but the same was not systematically controlled.

In contrast, our study indicates that smaller sheaths (14–15 Fr) are associated with improved safety profiles, including lower complication rates, decreased bleeding and more favourable recovery, albeit with reduced efficiency in managing complex stone burdens. Strategic preoperative planning with a CT scan, as implemented in our study, facilitates appropriate sheath selection. Preoperative stone burden scoring using GSS and post‐operative residual fragment determination via a CT scan have become standards of care for PCNL.^24^, ^25^ Our findings suggest that, in contemporary practice, surgeons are increasingly willing to adopt these strategies to optimize and report outcomes, even in the context of SM‐PCNL. Moreover, our study also did not include IRP measurements, which could provide important insights into the physiological effects of different sheath sizes. Finally, this study lacks a non‐suctioning arm, but previous studies demonstrated that SM‐PCNL was associated with shorter operative time and postoperative infectious complications.^26^

All the above limitations reduce the strength of causal inferences that can be drawn from the comparative analysis, highlighting the need for future randomized controlled trials to definitively determine optimal sheath selection strategies in SM‐PCNL.

## 5 CONCLUSIONS

This study shows that the decision of sheath size in SM‐PCNL is not a one‐size‐fits‐all approach. Intermediate sheath sizes (16–18 Fr) are typically utilized for cases involving larger stone volumes or challenging locations. Smaller sheaths (<16 Fr) are generally employed for less complex cases with lower stone burdens. Larger sheaths (20–22 Fr) may offer advantages for zero residual fragments in complex situations, but their use can be associated with an increased risk of complications. Adding suction does not necessarily reduce the invasiveness of PCNL. We suggest surgeons to consider stone burden and case complexity when selecting sheath size, and future randomized controlled trials may help determine whether 16 Fr and 18 Fr sheaths represent an optimal compromise.

## AUTHOR CONTRIBUTIONS

**Vineet Gauhar:** Conceptualization; data curation; writing—review and editing; supervision. **Bhaskar Somani:** Writing—review and editing. **Kemal Sarica:** Supervision. **Marek Zawadzki:** Data curation. **Abhishek Singh:** Data curation. **Khi Yung Fong:** Formal analysis. **Angelo Cormio:** Data curation. **Wei Zhu:** Data curation. **Jaisukh Kalathia:** Data curation. **Nariman Gadzhiev:** Data curation. **Vigen Malkhasyan:** Data curation. **Yadgar Shwani:** Data curation. **Oriol Angerri Feu:** Writing—review and editing. **Ben H. Chew:** Writing—review and editing. **Guohua Zheng:** Supervision. **Thomas R. W. Herrmann:** Writing—review Mand editing. **Daniele Castellani:** Conceptualization; formal analysis; writing—original draft.

## FUNDING/SUPPORT

None.

## CONFLICT OF INTEREST STATEMENT

Daniele Castellani certifies that all conflicts of interest, including specific financial interests and relationships and affiliations relevant to the subject matter or materials discussed in the manuscript (e.g., employment/affiliation, grants or funding, consultancies, honoraria, stock ownership or options, expert testimony, royalties or patents filed, received or pending), are the following: The authors declare no conflict of interest.
